# Eriodictyol as a Potential Candidate Inhibitor of Sortase A Protects Mice From Methicillin-Resistant *Staphylococcus aureus*-Induced Pneumonia

**DOI:** 10.3389/fmicb.2021.635710

**Published:** 2021-02-18

**Authors:** Li Wang, Qianxue Li, Jiaxin Li, Shisong Jing, Yajing Jin, Lin Yang, Hangqian Yu, Dacheng Wang, Tiedong Wang, Lin Wang

**Affiliations:** ^1^College of Animal Science, Jilin University, Changchun, China; ^2^Key Laboratory of Zoonosis Research, Ministry of Education, College of Veterinary Medicine, Jilin University, Changchun, China; ^3^Key Laboratory of Jilin Province for Zoonosis Prevention and Control, Institute of Military Veterinary Science, Academy of Military Medical Science, Academy of Military Science, Changchun, China

**Keywords:** antivirulence, eriodictyol, inhibitor, methicillin-resistant *Staphylococcus aureus*, sortase A, pneumonia

## Abstract

New anti-infective approaches are urgently needed to control multidrug-resistant (MDR) pathogens, such as methicillin-resistant *Staphylococcus aureus* (MRSA). Sortase A (SrtA) is a membrane-bound cysteine transpeptidase that plays an essential role in the catalysis of covalent anchoring of surface proteins to the cell wall of *Staphylococcus aureus* (*S. aureus*). The present study reports identification of a flavonoid, eriodictyol, as a reversible inhibitor of SrtA with an IC_50_ of 2.229 ± 0.014 μg/mL that can be used as an innovative means to counter both resistance and virulence. The data indicated that eriodictyol inhibited the adhesion of the bacteria to fibrinogen and reduced the formation of biofilms and anchoring of staphylococcal protein A (SpA) on the cell wall. The results of fluorescence quenching experiments demonstrated a strong interaction between eriodictyol and SrtA. Subsequent mechanistic studies revealed that eriodictyol binds to SrtA by interacting with R197 amino acid residue. Importantly, eriodictyol reduced the adhesion-dependent invasion of A549 cells by *S. aureus* and showed a good therapeutic effect in a model of mouse pneumonia induced by *S. aureus*. Overall, the results indicated that eriodictyol can attenuate MRSA virulence and prevent the development of resistance by inhibiting SrtA, suggesting that eriodictyol may be a promising lead compound for the control of MRSA infections.

## Introduction

Methicillin-resistant *Staphylococcus aureus* (MRSA) is a multidrug-resistant (MDR) bacterium resistant not only to methicillin but also to other β-lactam antibiotics (e.g., oxacillin or cefoxitin) and members of other antibiotic families (Jaradat et al., 2020). MRSA is an important human commensal and opportunistic pathogen that causes a broad spectrum of human diseases ranging from moderate skin infections to more severe endocarditis, bacteremia, sepsis, osteomyelitis, blood infections, and pneumonia (Diekema et al., 2001). Although the incidence rate of MRSA has been declining in many countries in recent years, high mortality rate associated with MRSA remains a great threat in clinical practice (Turner et al., 2019) and imposes a considerable economic burden (Zhen et al., 2020). The development of novel antibiotics remains an indispensable strategy for the treatment of MRSA infections; however, the development of new antibiotics is difficult, slow, and far behind the development of drug-resistant strains. Therefore, it is imperative to identify alternative strategies to combat increasingly powerful and evolving bacteria (Kali, 2015; Boswihi and Udo, 2018).

*Staphylococcus aureus* utilizes a vast array of virulence factors to survive and thrive in either normal host environments or under extreme conditions (Smeltzer, 2016), including surface-associated adhesins and secreted proteinaceous toxins. The secreted proteinaceous toxins and enzymes can damage host cells and tissues, are involved in the clearance of the host immune system, and promote bacterial spreading in the host (Oliveira et al., 2018). Targeting *S. aureus* virulence by small molecule inhibitors is a promising approach because it disarms bacteria to ameliorate infections but does not exert selective pressure on the bacteria thus reducing the risk of the development of antibiotic resistance (Clatworthy et al., 2007; Cegelski et al., 2008). Several surface-associated adhesins, such as ClfA/ClfB, fibronectin-binding protein (FnBPs) and collagen adhesin (CAN), are conducive to the pathogenesis of *S. aureus* infection (Patti et al., 1994; Que et al., 2001; Heying et al., 2007). These essential virulence factors of *S. aureus* are covalently anchored to the bacterial surface by sortase A (SrtA) (Geoghegan and Foster, 2017).

Sortase A is an extensively investigated membrane-localized transpeptidase that mediates the sorting of many adhesins to the bacterial surface and plays a key role in the pathogenesis of *S. aureus* infections (Maresso and Schneewind, 2008). SrtA is an extensively investigated membrane-localized transpeptidase that mediates the sorting of many adhesins to the bacterial surface and plays a key role in the pathogenesis of *S. aureus* infections (Jonsson et al., 2002, 2003; Bubeck Wardenburg et al., 2007). SrtA is not required for microbial growth and viability; thus, inhibition of SrtA is likely to impose a lower selection pressure on the development and spreading of the resistance mechanisms (Suree et al., 2007; Hou et al., 2018). Therefore, SrtA is considered an ideal druggable target for the development of novel anti-infective drugs.

Several classes of SrtA inhibitors, such as natural products, components of small molecule synthetic libraries, and peptidomimetics, have been shown to exert therapeutic effects on *S. aureus* infections *in vivo* (Cascioferro et al., 2014; Chung et al., 2019). Many natural products from microorganisms and plants have been reported as the candidate antivirulence agents against *S. aureus* and showed specific activity and high safety (Wu et al., 2019). The results of our experiments indicated that eriodictyol, an antioxidant flavonoid present in citrus fruits, is a reversible inhibitor of *S. aureus* SrtA. Eriodictyol can protect the cells from oxidative stress and radiation, has expectorant and other effects, and is extensively used as a clinical drug (Johnson et al., 2009; Bucolo et al., 2012; He et al., 2019). The inhibitory mechanisms were investigated, and the therapeutic effect of eriodictyol on MRSA-induced pneumonia was further evaluated in a mouse model. Our results demonstrated that eriodictyol can attenuate the virulence of *S. aureus* by inhibiting SrtA and that the administration of eriodictyol blocks the development of infection in a mouse pneumonia model. Therefore, eriodictyol can be potentially developed in the future as a therapeutic agent against *S. aureus* infections.

## Materials and Methods

### Bacterial Strains, Plasmids, and Culture Conditions

The MRSA strain USA300 used throughout this study was obtained from the American Type Culture Collection (ATCC, Manassas, VA, United States), and the SrtA deletion mutant (Δ*srtA*) was kindly provided by Dr. Xuming Deng. Clinically isolates of the MRSA strains SA28 and SA34 obtained within the past 3 years were randomly selected from our collection and were identified by 16S RNA and quality control. *Escherichia coli* (*E. coli*) DH5α was used as the bacterial host for the construction of the expression vectors. *S. aureus* and *E. coli* were grown in brain heart infusion (BHI, Hopebio, Qingdao, China) or Luria-Bertani broth (LB, Hopebio, Qingdao, China), respectively, in an incubator with shaking at 220 rpm at 37°C. When necessary, kanamycin (100 μg/mL) was added to the medium for plasmid selection or maintenance.

### Cloning and Preparation of Recombinant SrtA

The sequences encoding SrtA were amplified from *S. aureus* genomic DNA; the amplified fragment was treated with *Nde*I and *Bam*HI and then inserted into the cloning site of the *Nde*I/*Bam*HI-digested pET28a to yield pET28a-SrtA. Site-directed mutagenesis of A92L, A104L, I182A, and R197A was conducted using a recombinant vector encoding SrtA and a Mut Express MultiS fast mutagenesis kit (Vazyme Biotech, Nanjing, China). All primers used in the study are shown in Table 1. The pET28a-SrtA and mutant constructs were transformed into the *E. coli* overexpression strain BL21 (TianGen, Beijing, China) to express the SrtA proteins. The expression of recombinant proteins was induced overnight with 0.5 mM isopropyl-ß-D-thiogalactoside (IPTG) at 16°C, and the proteins were purified using a HIS-Select nickel affinity gel (Beyotime, Shanghai, China) system as reported previously (Lu et al., 2007).

**TABLE 1:** Primers used in this study.

Primer name	Sequences (5′-3′)
*srtA*-F	GGGAATTCCATATGCAAGCTAAACCTCAAATTCCG
*srtA*-R	CGCGGATCCTTATTTGACTTCTGTAGCTACAAAGA
A92L-*srtA*-F	GACCAAAAACACCTGAACAATTAAA
A92L-*srtA*-R	CTGGATATACTGGTTCTTTAATATCAGC
A104L-*srtA*-F	CTTTAAAGAAGAAAATGAATCACTA
A104L-*srtA*-R	CTTACACCTCTATTTAATTGTTCAG
I182A-*srtA*-F	AACATTAGCTACTTGTGATGATTAC
I182A-*srtA*-R	AATTGTTTATCTTTACCTTTTTGTTCA
R197A-*srtA*-F	GGAAAAAGCTAAAATCTTTGTAGCT
R197A-*srtA*-R	CAAACGCCTGTCTTTTCATTG

### SrtA Activity Measurement

Sortase A activity was measured by the fluorescence resonance energy transfer (FRET) assay as described previously (Ton-That et al., 1999; Mazmanian et al., 2002). Briefly, the assay was performed in 300 μL of the reaction system in 96-well black plates containing 10 μM recombinant SrtA and various concentrations of eriodictyol for 30 min at 37°C; then, the SrtA substrate peptide Abz-LPATG-Dap (Dnp)-NH_2_ was added to a final concentration of 50 μM. The plates were incubated for 1 h at room temperature. Wells containing all components without SrtA were used as negative controls. Fluorescence was quantified at an excitation wavelength of 309 nm and an emission wavelength of 420 nm. The IC_50_ values were calculated by GraphPad Prism software. Three biological replicates were assayed for each test, and the data were expressed as the mean ± SEM.

### Reversible Inhibition Assay

Reversible inhibition of SrtA was assayed as described in a previous report (Zhang et al., 2014). Briefly, 100 μL of SrtA was mixed with eriodictyol at a concentration of 22.290 μg/mL (10-fold IC_50_). After incubation for 1 h at room temperature, the mixture was diluted 100-fold by adding 9.9 mL of reaction buffer. Then, 10 μL (200 μM) of the fluorescent substrate peptide was mixed with 190 μL of the diluted mixture. The fluorescence intensity measurements were performed at 309 and 420 nm excitation and emission wavelength, respectively. The experiment was carried out in three biological replicates, and the data were expressed as the mean ± SEM.

### Susceptibility Testing and Growth Curve Assay

The broth microdilution method was used to determine the minimal inhibitory concentrations (MICs) according to the NCCLS guidelines. The details of susceptibility testing and growth curve determination are described in the Supplementary Material.

### Cytotoxicity Assay

Cytotoxicity was determined using a cell counting kit-8 (CCK-8, Transgen, Beijing) as described previously (Riss et al., 2011). Briefly, 96-well culture plates were seeded with 100μL/well of a suspension of Vero cells (5 × 10^4^ cells per well) and incubated at 37°C in an atmosphere of 5% CO_2_ for 24 h. Then, various concentrations of eriodictyol (0–512 μg/mL) were added, and the incubation was continued for 24 h. Then, 10 μL of CCK-8 was added to each well, and the plate was incubated for 1–4 h; the absorbance was measured at 450 nm. The experiment was repeated at least three times, and the curve of the eriodictyol concentration versus the cell percentage inhibition rate (1-cell viability) was plotted.

### Adherence of *S. aureus* to Immobilized Fibrinogen

*Staphylococcus aureus* USA 300 and clinical isolates SA28 and SA34 were grown overnight and diluted 1:100 in fresh BHI with or without eriodictyol; then, the bacteria were incubated at 37°C and 180 rpm until A_600__nm_ reached 0.5. Bacterial culture (100 μL) was added to a polystyrene Costar 96-well plate precoated with 20 μg/mL bovine fibrinogen at 4°C overnight. After incubation at 37°C for 2 h, the bacterial suspension was discarded, and the wells were washed twice with PBS. Then, the cells adherent to the bottom of the plates were fixed with 25% formaldehyde for 30 min. The plate was washed twice with PBS; the cells were stained with crystal violet for 20 min, and the absorbance at 570 nm was read using a microplate reader. The Δ*srtA* strain was treated under the same conditions and used as a positive control.

### Internalization Assay

The effect of eriodictyol on the internalization of *S. aureus* USA 300 and clinical isolates SA28 and SA34 into the human lung adenocarcinoma cell line A549 (ATCC; CCL-185) was determined as described previously (Alva-Murillo et al., 2012; Truong-Bolduc et al., 2015). In brief, A549 cells (3 × 10^5^ cells per well) were seeded in a 24-well plate and cultured in an atmosphere of 5% CO_2_ at 37°C for 24 h. *S. aureus* USA300 was cultured in BHI broth supplemented with various concentrations of eriodictyol (64, 128, or 256 μg/mL) or 0.5% DMSO at 37°C to reach A_600__nm_ = 1.0. The Δ*srtA* strain cultured in BHI broth was used as a positive control. The cells were mixed with *S. aureus* at a multiplicity of infection (MOI) of 10:1 (10^6^ washed bacteria/10^5^ cells) and incubated for 2 h at 37°C at 5% CO_2_. The wells were washed three times with PBS (pH 7.4) to remove non-adherent bacteria and then incubated for 1 h at 37°C at 5% CO_2_ in the medium containing 200 μg/mL gentamicin to eradicate the extracellular bacteria. The cells were washed and lysed with sterile distilled water; the lysate was diluted 100-fold with PBS and spread on BHI agar plates in triplicate. The colony-forming units (CFUs) were manually counted, and the data were processed according to the method described by Cheung and Bayles (2007).

### Crystal Violet Biofilm Assay

An overnight bacterial culture was diluted 1:100 in BHI media containing various concentrations of eriodictyol ranging from 32 to 256 μg/mL and grown with shaking at 37°C to an A_600__nm_ of 0.6. Then, 5 μL of the bacterial culture was added to 195 μL of BHI broth enriched with 1% glucose to form a biofilm at 37°C for 18 h. Determination and quantitative analysis of the biofilm were performed as described previously (Merritt et al., 2011).

### Staphylococcal Protein A Display Analysis

Staphylococcal protein A (SpA) analysis was performed as described in a previous study (Kong et al., 2015). Briefly, *S. aureus* USA 300 was cultured at 37°C with shaking with or without eriodictyol to an A_600__nm_ of 1.0. Then, 50 μL of bacterial solution was incubated with an equal volume of a 1:50 dilution of FITC-labeled rabbit anti-goat-IgG (Sigma, United States) in the dark at 37°C for 1 h. After washing and resuspending in PBS, 10 μL of the solution of stained bacteria was dropped onto a microscope slide and covered with a cover slip. Finally, the distribution of SpA on the cell surface was evaluated using a laser confocal fluorescence microscopy.

### Western Blot Analysis

*Staphylococcus aureus* strain USA 300 was cultured in BHI broth containing different concentrations of eriodictyol (0, 32, 64, or 128 μg/mL) until an A_600__nm_ of 0.8 was reached. The total protein of *S. aureus* buds was extracted in lysis buffer (10 mM Tris–HCl, 1 mM EDTA, and 250 mM sucrose, pH 7.4) supplemented with 10 mg/mL lysozyme and 40 mg/mL lysostaphin. Equal amounts of bacterial proteins (20 μg) were separated by 12% SDS-PAGE and then transferred onto polyvinylidene difluoride (PVDF) membranes. After blocking with 5% (w/v) non-fat milk for 1 h, the membranes were washed three times with TBST (TBS with 0.05% Tween 20) and then incubated with polyclonal antiserum against SrtA (1:5000) at room temperature for 2 h. The membranes were washed three times with TBST and hybridized with horseradish peroxidase (HRP)-conjugated secondary goat anti-rabbit secondary antibody at room temperature for 1 h. Finally, immunoreactive bands were visualized with ECL substrate (Beyotime, China) on a ECL detection system (GE Healthcare, United Kingdom).

### Fluorescence Quenching Assay

The binding constant (*K*_*A*_) of eriodictyol to SrtA was measured by the fluorescence quenching assay as described previously (Papadopoulou et al., 2005). Eriodictyol solution (10 μL) was mixed with 990 μL of purified SrtA (5 μM) containing eriodictyol (0, 2, 4, 6, 8, 12, 14, 16, and 18 μg). The excitation wavelength was set at 280 nm with a 5-nm bandpass filter, and the emission slit width was 10 nm. The fluorescence emission spectrum of the mixed solution (260–400 nm) was recorded by a fluorescence spectrophotometer (RF5301, Japan), and all measurements were repeated in triplicate. The fluorescence quenching data were plotted as the relative fluorescence intensity (RFI = *F/F_0_* × 100) versus eriodictyol concentration to generate the Stern-Volmer plot of *F_0_/F* versus [*Q*]; the *K*_*A*_ values were calculated by linear regression. The details of the measurements were reported previously (Kim et al., 2006).

### Molecular Modeling

Details of molecular modeling are described in the Supplementary Material.

### Mouse Model of *S. aureus* Pneumonia

Inbred C57BL/6J mice 7 weeks of age were obtained from the Experimental Animal Center of Jilin University. Animal experiments were carried out according to the ethical standards and protocols approved by the Institutional Animal Care and Use Committee (IACUC) of Jilin University. *S. aureus* bacteria were grown overnight with shaking at 37°C and diluted 1:100 into 200 mL of fresh sterile BHI broth to grow to an *A*_600 nm_ = 1.0. Then, culture aliquots (10 mL) were collected by centrifugation; the bacterial sediments were washed three times with PBS and suspended in 100 μL of PBS. A mouse model of *S. aureus*-induced pneumonia was established as described previously (Qiu et al., 2012). In brief, the mice were anesthetized with vaporized isoflurane and inoculated with 30 μL of *S. aureus* suspension to deliver 2 × 10^8^ CFU of bacteria via the intranasal route. After intranasal inoculation, the mice were held upright for 2 min and subcutaneously injected with 100 mg/kg eriodictyol 1 h after the infection. Subsequently, the mice were treated with eriodictyol at 12 h intervals. The mice in the control group were subcutaneously administered sterile PBS containing 0.5% DMSO (mock-treated) on the same schedule. Survival of the mice was assessed every 12 h for 96 h after the administration to evaluate the percentage of survival.

For evaluation of bacterial count in the lung tissue and histopathological analysis, the mice were infected with 30 μL (1 × 10^8^ CFU) of *S. aureus* suspension and sacrificed by cervical dislocation 24 h after the infection. Then, the lung was excised using aseptic techniques and homogenized; the staphylococcal burden was determined by spotting serial dilutions of the homogenate in 0.9% saline on BHI agar plates. The right lung of the mice was removed and perfused with PBS. Then, formalin-fixed tissues were routinely processed and stained with hematoxylin and eosin (H&E); lung sections were imaged by light microscopy.

## Results

### Identification of Eriodictyol as an Inhibitor of SrtA

**FIGURE 1:**
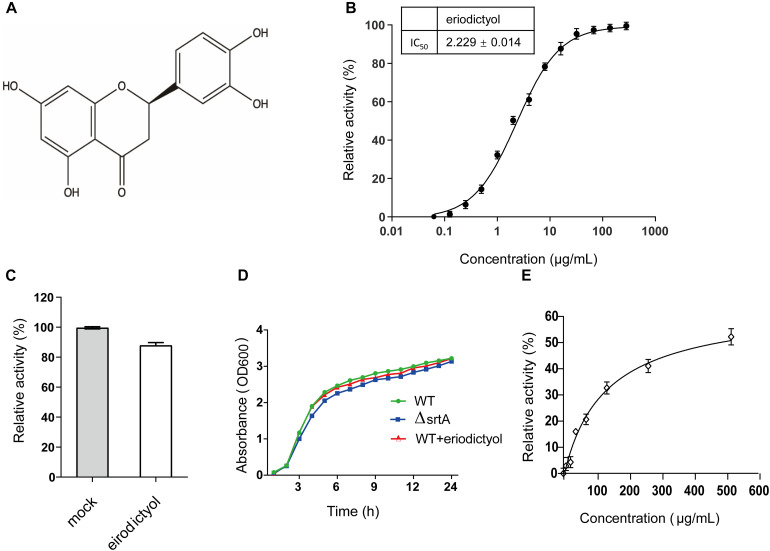
Eriodictyol acts as a reversible inhibitor of SrtA. **(A)** Chemical structure of eriodictyol. **(B)** Eriodictyol inhibited the cleavage of the Abz-LPATG-Dap (Dnp)-NH_2_ substrate by SrtA in a dose-dependent manner. Each reaction condition was assayed in triplicate, and the data are presented as the mean ± SEM. **(C)** SrtA was treated with or without 10 × IC_50_ of eriodictyol and then diluted; the SrtA activity was subsequently determined by FRET assay. Untreated SrtA (mock) was assumed as 100% activity. **(D)** Growth curves of *S. aureus* USA 300 and the Δ*srtA* mutant strains with or without eriodictyol (128 μg/mL). **(E)** Percentage of viability of Vero cells was measured by the CCK-8 assay after 24 h of incubation with eriodictyol (0–512 μg/mL).

The inhibitory effect of eriodictyol on the activity of SrtA was evaluated using the FRET assay based on the changes in the fluorescence signal of the peptide Abz-LPATG-Dap (Dnp)-NH_2_ containing the SrtA recognition motif (Oh et al., 2006). Screening identified eriodictyol (Figure 1A), a flavonoid, with high inhibitory activity against *S. aureus* SrtA with an IC_50_ value of 2.229 ± 0.014 μg/mL (Figure 1B). To evaluate whether the inhibition of SrtA by eriodictyol was reversible, SrtA was mixed with eriodictyol at a 10-fold IC_50_ concentration for 1 h, diluted 100-fold, and incubated with the substrate peptide. The recovery of the activity of SrtA was 87.839 ± 1.908% compared with that in the control group after the dilution (Figure 1C). These results indicated that eriodictyol functions as a reversible inhibitor of SrtA, which non-covalently interacts with the active site of SrtA. The MIC of eriodictyol against *S. aureus* USA 300 was greater than 512 μg/mL, and the growth curves of *S. aureus* USA 300 treated with or without 128 μg/mL eriodictyol showed a similar growth pattern (Figure 1D). These results demonstrated that eriodictyol did not inhibit the growth of *S. aureus* even at a concentration 50-fold higher than IC_50_. The CCK-8 assay was subsequently used to evaluate the cytotoxicity of eriodictyol in Vero cells; the results showed that 80% of the cells remained viable after the treatment with a concentration 30-fold higher than IC_50_ (Figure 1E). Thus, eriodictyol was identified as a potential small molecule inhibitor of SrtA that is effective and safe at a concentration considerably lower than MIC.

### Eriodictyol Inhibits the Adhesion of *S. aureus* to Fibrinogen

**FIGURE 2:**
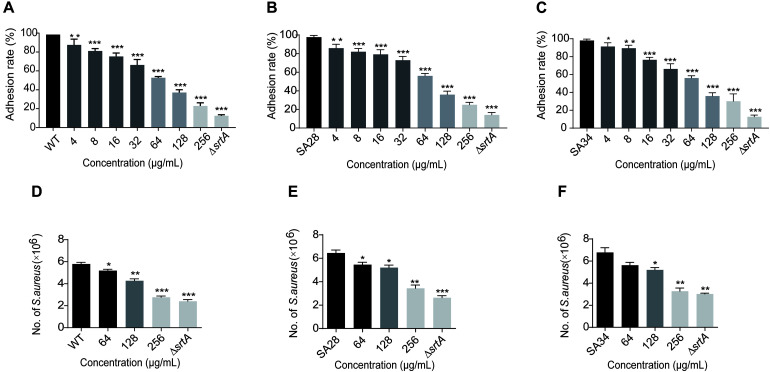
Eriodictyol inhibition on the adherence and invasion of *S. aureus* USA 300 and clinical isolates. **(A–C)** The effect of eriodictyol on the adhesion of *S. aureus* USA 300 and clinical isolates SA28 and SA34 to fibrinogen. **(D–F)** Eriodictyol suppressed the internalization of *S. aureus* USA 300 and clinical isolates SA28 and SA34 into A549 cells. A549 cells were infected with *S. aureus* pretreated with various concentrations of eriodictyol and lysed 2 h after the infection; the number of viable *S. aureus* in the cells was quantified by serial dilution on LB agar plates. The data are shown as the mean ± SEM (error bars) of three replicates. * *P* < 0.05, ** *P* < 0.01, *** *P* < 0.001 vs. the WT group according to the two-tailed Student’s *t*-test.

Binding to fibronectin and fibrinogen is important for the pathogenesis of *S. aureus*. *S. aureus* with deletion of the SrtA gene does not display clumping factors (ClfA and ClfB) and fibronectin-binding proteins A and B (FnbA and FnbB), resulting in attenuated virulence (Oh et al., 2006). Therefore, we hypothesized that a SrtA inhibitor reduces the adhesion of *S. aureus* to fibrinogen. As shown in Figure 2A, increasing eriodictyol concentrations (from 4 to 256 μg/mL) induced gradually increasing inhibition of the adhesion of *S. aureus* USA 300 to fibrinogen. Comparison with the wild-type (WT) control group indicated that treatment with 256 μg/mL eriodictyol suppressed the adhesion of WT to fibrinogen down to 22.895 ± 1.507%. Similar results were observed in the clinical isolates SA28 and SA34 of *S. aureus*; treatment with 256 μg/mL eriodictyol reduced the adhesion of the clinical strains SA28 and SA34 to fibrinogen down to 25.095 ± 0.697% and 30.010 ± 1.012%, respectively (Figures 2B,C). Minimum fibrinogen-binding capacity of the Δ*srtA* strain was 12.510 ± 0.472%.

### Eriodictyol Suppresses the Internalization of *S. aureus* Into A549 Cells

Surface proteins, such as FnBPs and IsdB, are involved in promoting bacterial invasion into epithelial and endothelial cells and are covalently anchored to the cell wall envelope by SrtA (Foster et al., 2014). Epithelial cells are the usual initial site of *S. aureus* infection; hence, colonization on the cell surface and invasion of the cells via SrtA-mediated cell surface proteins may result in acute and chronic infections (Gómez et al., 2004). We investigated whether eriodictyol inhibits the internalization of *S. aureus* USA 300, SA28, and SA34 into A549 lung epithelial cells. As shown in Figures 2D–F, treatment of *S. aureus* USA 300 and clinical isolates SA28 and SA34 with 256 μg/mL eriodictyol reduced the number of bacteria entering the cells compared to that in the PBS-treated control.

### Eriodictyol Inhibits the Formation of the Biofilm of *S. aureus*

**FIGURE 3:**
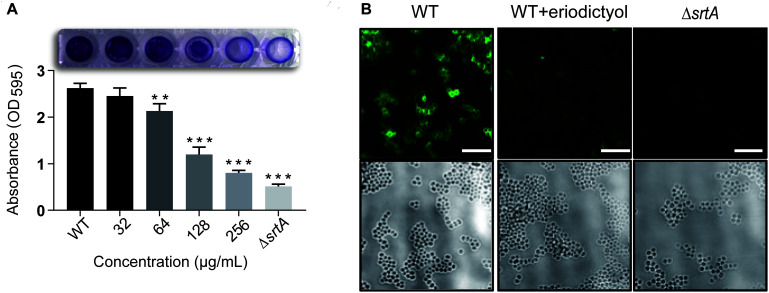
Eriodictyol inhibits SrtA activity *in vitro*. **(A)** Biofilm formation of *S. aureus* in the presence of eriodictyol. The formation of the biofilms was quantified by the crystal violet (CV) staining assay. **(B)** Confocal laser microscopy analysis of *S. aureus* surface protein (SpA) stained with FITC-labeled rabbit IgG. Magnification: 600×; scale bar, 50 μm. The data are shown as the mean ± SEM (error bars) of three replicates. ** *P* < 0.01, *** *P* < 0.001 vs. the WT group according to the two-tailed Student’s *t*-test.

Attachment to a surface is the first step in the formation of *S. aureus* biofilms. Previous studies demonstrated that a series of specific staphylococcal surface proteins known as MSCRAMMs (microbial surface components recognizing adhesive matrix molecules) play an important role in the interaction with human matrix proteins. MSCRAMMs are covalently anchored to bacterial cell wall peptidoglycans by SrtA (Geoghegan and Foster, 2017). These considerations suggested that eriodictyol may reduce the formation of the biofilms. To verify this hypothesis, we used a biofilm assay, in which the biofilm biomass was quantified by measuring the absorbance after staining with crystal violet. As shown in Figure 3A, the treatment of *S. aureus* with various concentrations of eriodictyol decreased the biofilm biomass in a dose-dependent manner. Treatment with 128 μg/mL eriodictyol inhibited the biofilm biomass by 80.380 ± 0.034% compared with that in the WT control group. This result suggests that eriodictyol can reduce the formation of the biofilms by inhibiting SrtA activity.

### Eriodictyol Inhibits the Anchoring of Surface Protein A in *S. aureus*

One of the surface proteins anchored by SrtA, SpA, specifically binds to the Fc region of IgGs and plays an essential role in the pathogenicity of *S. aureus* infection (Aybay, 2003). Previous studies demonstrated that SpA is important for an increase in inflammation of the pulmonary epithelium in *S. aureus* pneumonia and enhances the survival of bacteria in the bloodstream in renal abscess (Cheng et al., 2009) and septic arthritis (Palmqvist et al., 2002). Thus, we evaluated the inhibitory effect of eriodictyol on SpA in the cell wall by staining *S. aureus* cells with FITC-labeled goat anti-rabbit IgG. The confocal microscopy images showed the absence of fluorescence in the Δ*srtA* group and a significant decrease in the fluorescence intensity in the 256 μg/mL eriodictyol-treated group compared with that in the untreated group (Figure 3B).

### Eriodictyol Has No Effect on the Expression of SrtA

**FIGURE 4:**
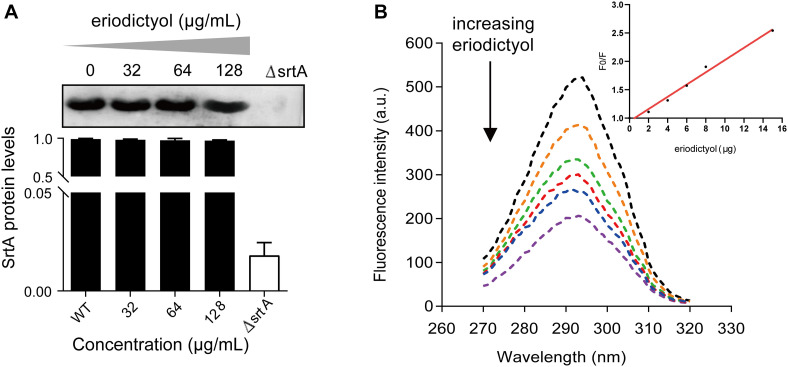
The expression level of SrtA and the interaction between eriodictyol and StrA measured by the fluorescence quenching assay. **(A)** Western blot analysis of SrtA from *S. aureus* treated with various concentrations of eriodictyol and grayscale analysis of the SrtA protein bands. **(B)** Fluorescence quenching assays were performed to evaluate the binding affinity of eriodictyol to SrtA.

Based on these results, we aimed to determine whether the inhibition of SrtA activity by eriodictyol was due to the inhibition of SrtA protein expression. Bacteria were cultured overnight in the presence of various concentrations of eriodictyol (0, 32, 64, or 128 μg/mL), and the total proteins of *S. aureus* were extracted. The levels of SrtA expression were detected by Western blotting, and grayscale image analysis was used to quantify the data. As shown in Figure 4A, eriodictyol did not interfere with the expression of SrtA.

### Investigation of the Interaction of Eriodictyol With SrtA by Fluorescence Quenching

Fluorescence quenching analysis was used to determine the binding affinity between eriodictyol and SrtA. As illustrated in Figure 4B, a gradual increase in the concentration of eriodictyol resulted in quenching of the fluorescence of SrtA in a dose-dependent manner, and the effect of eriodictyol concentration on the fluorescence intensity was linear (Figure 4B, inset). We further calculated that the binding constant *K*_*A*_ of SrtA to eriodictyol was 8.2 × 10^4^L/mol, suggesting a significant binding interaction between eriodictyol and SrtA.

### Determination of the Molecular Mechanism

**FIGURE 5:**
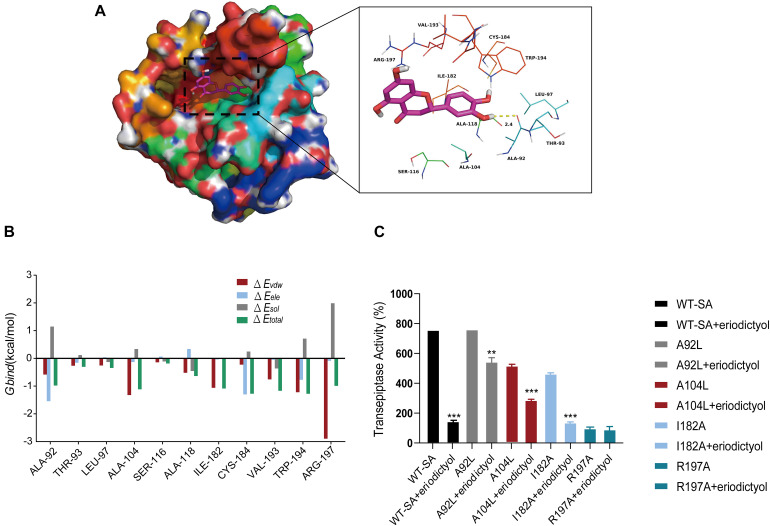
Molecular modeling of the interaction of eriodictyol with SrtA. **(A)** Decomposition of the free energy on a per-residue basis for the binding of eriodictyol to modeled *S. aureus* SrtA. **(B)** Docking model of eriodictyol within SrtA generated by molecular dynamics simulation. **(C)** WT-SrtA and SrtA mutants (A92L-SrtA, A104-SrtA, I182A-SrtA, and R197A-SrtA) were incubated with 64 μg/mL eriodictyol, and the transpeptidase activity of recombinant SrtA was determined by FRET. ** *P* < 0.01, *** *P* < 0.001 compared with the WT group.

Molecular modeling studies were performed to determine the mechanism of inhibition of SrtA by eriodictyol. The Ala-92 residue had a strong electrostatic (Δ*E*_*ele*_) contribution to the formation of the SrtA-eriodictyol complex with a Δ*E*_*ele*_ of <−1.5 kcal/mol (Figure 5A). Detailed analysis showed that the Ala-92 residue participated in a strong hydrogen bond interaction with the oxygen atom of the hydroxyl group of eriodictyol with a bond length of 2.4 Å (Figure 5B). Additionally, the Arg-197 residue was involved in the strong van der Waals interactions (Δ*E*_*vdw*_ of <−2.5 kcal/mol) with the ligand because of the close proximity of the residue and eriodictyol. Overall, the majority of the decomposed energy interactions originated from the van der Waals interactions mainly due to hydrophobic interactions involving Leu-97, Ala-104, Ala-118, Ile-182, Val-193, and Trp-194. The total free binding energy of the SrtA-eriodictyol complex was estimated as Δ*G*_*bind*_ of −12.4 kcal/mol for eriodictyol, suggesting that eriodictyol can bind to the binding site of SrtA. Overall, molecular modeling provided a reasonable explanation for the interactions between eriodictyol and SrtA, and this information is valuable for the development of SrtA inhibitors in the future.

The binding model of eriodictyol and SrtA guided our site-directed mutagenesis study. The mutations were generated, and FRET experiments were performed to measure the transpeptidase activity of SrtA and its mutants, including A92L-SrtA, A104L-SrtA, I182A-SrtA, and R197A-SrtA, in the presence of eriodictyol (64 μg/mL). As shown in Figure 5C, inhibition of the activity of SrtA mutants (A92L-SrtA, A104L-SrtA, and I182A-SrtA) by eriodictyol was significantly reduced, and the R197A mutant of SrtA lost transpeptidase activity.

### Eriodictyol Protects Mice From *S. aureus* Pneumonia

**FIGURE 6:**
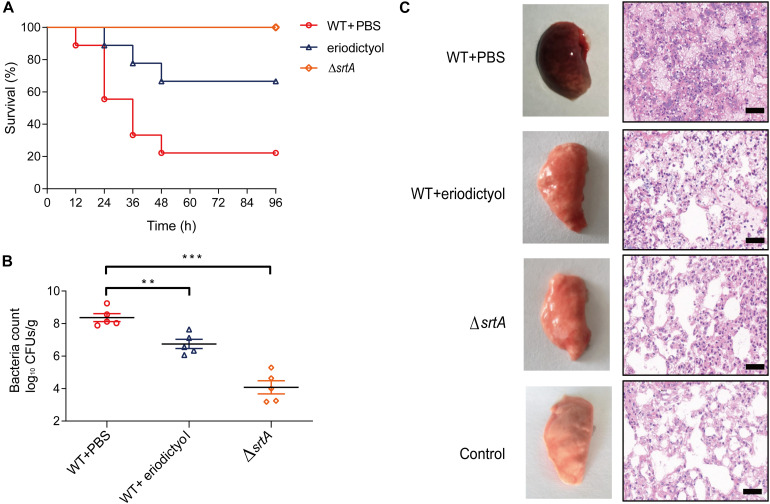
Eriodictyol protects the mice from *S. aureus*-induced pneumonia. **(A)** Effect of eriodictyol treatment on the survival of mice (*n* = 10) infected with a lethal dose of *S. aureus*. Mock vs. eriodictyol, **P < 0.01; log-rank test. **(B)** Effect of eriodictyol treatment (100 mg/kg) on the bacterial load in the lungs of mice (*n* = 5). **P < 0.01, ***P < 0.001 vs. the WT group; Mann-Whitney test, two-tailed. Horizontal bars represent the mean values. **(C)** Histopathology of the lung (H&E staining) of mice treated with or without eriodictyol (100 mg/kg/d). The animal data were obtained in two separate experiments (magnification: 400×; scale bar, 50 μm).

To investigate the *in vivo* activity of eriodictyol, we constructed a mouse model of *S. aureu*s-induced pneumonia. Groups of 7-week-old mice were intranasally (i.n.) inoculated with a lethal dose of *S. aureus* (2 × 10^8^ CFU/per mouse) and were treated with 100 mg/kg eriodictyol every 12 h. The mortality rate of infected animals was assessed at 12-h intervals for 96 h. As shown in Figure 6A, the percentage of mortality in the eriodictyol-treated group at 24, 48, and 72 h was significantly lower than that in the control group. Moreover, treatment with eriodictyol reduced the number of viable *S. aureus* in the lung tissues compared with that in the mock-treated group (administered PBS containing 0.5% DMSO) (Figure 6B).

Gross examination of lung tissues was used to evaluate the pathological relevance of eriodictyol treatment; the results showed that the lung tissues of eriodictyol-treated mice were pink and spongy and had less focal infection, whereas the lung tissues of untreated mice were red and superficially mottled and had many infectious foci. Histopathological analysis revealed that most of the alveolar spaces in the samples of the mock-treated mice were infiltrated by inflammatory cells, whereas treatment with eriodictyol significantly alleviated inflammation due to reduced accumulation of inflammatory cells in the alveolar spaces (Figure 6C). Overall, we conclude that eriodictyol attenuated the virulence of *S. aureus in vivo* and protected against *S. aureus*-induced pneumonia.

## Discussion

Methicillin-resistant *Staphylococcus aureus* is one of the most common pathogens of nosocomial pneumonia (Chastre et al., 2014). Recent studies reported a gradual increase in the proportion of MRSA isolated from patients with infectious pneumonia that leads to significant morbidity and mortality (Iwata et al., 2020). Traditionally, antibiotics have been used as the primary weapon against infections caused by *S. aureus*. Initially, antibiotics are highly effective; however, high selective pressure and improper use resulted in the emergence, spreading, and expanded prevalence of antibiotic-resistant bacteria. Identification of new therapeutic targets and novel strategies to combat *S. aureus*-related infections are thus urgently needed (Maresso and Schneewind, 2008; Zhang et al., 2014).

Sortase has been extensively characterized as an ideal target for the development of anti-infective drugs due to transpeptidation of many surface proteins, which are virulence factors required for *S. aureus* infection (Ton-That and Schneewind, 1999). Sortase substrates function as adhesins, internalins, and immune evasion factors and are involved in blood clotting and nutrient transport across the microbial cell wall envelope (Navarre et al., 1998). Inhibitors of SrtA can reduce the pathogenicity of bacteria to enable elimination of the bacteria by host immunity.

Natural compounds have become attractive anti-infection agents due to their safety and environmental friendliness recognized by long-term practice. Identification of new small molecules with antivirulence activities in bioactive natural products is a promising approach for the treatment of the diseases caused by MRSA. The FRET assay was used to screen for the inhibitors of SrtA because SrtA can recognize and cleave peptides with LPXTG motifs (Mazmanian et al., 2002). Eriodictyol was identified as a new SrtA inhibitor with an IC_50_ of 2.229 μg/mL at very low concentrations. The inhibitory activity of eriodictyol *in vitro* was significantly better than that of natural product inhibitors reported previously, such as berberine chloride (Kim et al., 2004), a bisindole alkaloid (Oh et al., 2005), and isoaaptamine (Jang et al., 2007). What is gratifying is that eriodictyol belongs to flavonoids without any reported biohazards. Cytotoxicity experiments showed that 80% of the Vero cells remained viable after the treatment with eriodictyol at a concentration 30-fold higher than IC_50_. High efficiency and low toxicity of eriodictyol suggests that this compound can be considered a promising candidate inhibitor of SrtA for follow-up investigation. Additionally, eriodictyol inhibited the virulence-related phenotype of SrtA, including the adhesion of the bacteria to fibrinogen, the formation of the biofilms, and the anchoring of SpA on the cell wall. Virtual docking results suggested that eriodictyol binds to the binding pocket of SrtA mainly due to the hydrogen bonding and electrostatic and *van der Waals* interactions. The binding model guided our mutagenesis study that systematically replaced each amino acid in the binding pocket of SrtA. FRET experiments confirmed that four mutated amino acids (A92, A104, I182, and R197) were involved in the binding of eriodictyol to SrtA. Arg197 is one of the three conserved amino acid residues in the active site of the enzymes of the sortase family. Mutation of Arg197 leads to the loss of the *in vivo* and *in vitro* transpeptidation activity of sortase (Marraffini et al., 2004), which is consistent with the results of our FRET experiments. Earlier studies demonstrated that SrtA mutants are defective in the display of surface proteins and exhibit lower virulence in animal models compared to that of WT bacteria (Mazmanian et al., 2000). We also investigated the protective effects of eriodictyol in a mouse model of MRSA infection-induced pneumonia. As expected, eriodictyol showed excellent protective effect against lethal pneumonia caused by *S. aureus.*

In conclusion, this study demonstrated that eriodictyol is a reversible inhibitor of SrtA and can attenuate MRSA virulence *in vivo*. Thus, eriodictyol could be a potential lead compound for further development and use as an antivirulence drug for the treatment of MRSA infections.

## Data Availability Statement

The original contributions presented in the study are included in the article/Supplementary Material, further inquiries can be directed to the corresponding author.

## Ethics Statement

The animal study was reviewed and approved by Institutional Animal Care and Use Committee (IACUC) of Jilin University.

## Author Contributions

QL, TW, and DW conceived and designed the experiments. LiW, JL, and SJ performed and analyzed the experiments. YJ, LY, and HY performed the molecular dynamics simulation. LiW prepared the original manuscript. LinW revised the manuscript. All authors contributed to the article and approved the submitted version.

## Conflict of Interest

The authors declare that the research was conducted in the absence of any commercial or financial relationships that could be construed as a potential conflict of interest.
