# Supplementary material for: Eriodictyol as a Potential Candidate Inhibitor of Sortase A Protects Mice From Methicillin-Resistant Staphylococcus aureus-Induced Pneumonia
Source: Front Microbiol. 2021 Feb 18;12:635710. doi: 10.3389/fmicb.2021.635710 (PMC7929976; doi:10.3389/fmicb.2021.635710)
Supplement: Supplementary file 1 [file Data_Sheet_1.PDF]

Supplementary Material

Supplementary Data

Susceptibility testing and growth curve assay

The broth microdilution method was performed to determine the minimal inhibitory concentrations (MICs) according to the NCCLS guidelines. Briefly, 96-well plates containing eriodictyol concentrations ranging from 2 to 1024 μg/mL and *S. aureus* USA300 (1×10^5 CFU) in 100 μL Cation-Adjusted Mueller Hinton broth (CAMHB, Hopebio, Qingdao, China) were incubated at 37°C for 18-22 h before the determination of absorbance. Experiments were repeated with at least three biological replicates. For growth curve assay, *S. aureus* USA300 was grown overnight and inoculated in fresh BHI (1:100) containing 128 μg/mL eriodictyol to continue culture until the Absorbance $(A)_{600nm}$ reached 3.0. The Δ*srtA* strain and USA300 without eriodictyol were used as control groups. The cell growth condition was recorded at A_{600nm} every 1 h.

Molecular modeling

In order to investigate the binding mechanism of Eriodictyol with SrtA, the SrtA structure from *S. aureus* (Protein Data Bank [PDB] ID: 1T2P) was used. The preparation of Eriodictyol and SrtA protein structures and the molecular docking was performed using default parameters in the AutoDock vina 1.1.2 package (Vina, 2010). The best docked pose (conformation) in the Eriodictyol-SrtA complex obtained from molecular docking was subject to 25 ns molecular dynamics simulations using the Amber14 software package (Götz et al., 2012; Salomon-Ferrer et al., 2013), the preparation of the complex, the protocol of the molecular dynamics simulation were performed as described previously (Niu et al., 2013).

The binding free energies (ΔG_{bind} in kcal/mol) were calculated using the Molecular Mechanics/Generalized Born Surface Area (MM/GBSA) method, implemented in AmberTools 15. Moreover, to identify the key protein residues responsible for the ligands binding process, the binding free energy was decomposed on a per-residue basis. For each complex, the binding free energy of MM/GBSA was estimated as follows:

$$\Delta G_{bind} = G_{complex} - G_{protein} - G_{ligand}$$

where ΔG_{bind} is the binding free energy and $G_{complex}$, $G_{protein}$ and G_{ligand} are the free energies of complex, protein, and ligand, respectively.

REFERENCES

Götz, A.W., Williamson, M.J., Xu, D., Poole, D., Le Grand, S., and Walker, R.C. (2012). Routine microsecond molecular dynamics simulations with AMBER on GPUs. 1. Generalized born. Journal of chemical theory and computation 8(5), 1542-1555. doi： 10.1021/ct200909j.

Niu, X., Qiu, J., Wang, X., Gao, X., Dong, J., Wang, J., et al. (2013). Molecular insight into the inhibition mechanism of cyrtominetin to α-hemolysin by molecular dynamics simulation. European journal of medicinal chemistry 62, 320-328.doi: 10.1016/j.ejmech.2013.01.008.

Salomon-Ferrer, R., Götz, A.W., Poole, D., Le Grand, S., and Walker, R.C. (2013). Routine microsecond molecular dynamics simulations with AMBER on GPUs. 2. Explicit solvent particle mesh Ewald. Journal of chemical theory and computation 9(9), 3878-3888 doi: 10.1021/ct400314y.

Vina, A. (2010). Improving the speed and accuracy of docking with a new scoring function, efficient optimization, and multithreading Trott, Oleg; Olson, Arthur J. J. Comput. Chem 31(2), 455-461 doi: 10.1002/jcc.21334.
